# The neural bases of frontotemporal dementia and primary progressive aphasia subtypes: insights from activation likelihood estimation meta-analyses of 8057 patients

**DOI:** 10.1093/braincomms/fcag223

**Published:** 2026-06-13

**Authors:** Zlatomira G Ilchovska, Justine Lockwood, Elizabeth Proctor, Akram A Hosseini, Matthew A Lambon Ralph, JeYoung Jung

**Affiliations:** School of Psychology, University of Nottingham, Nottingham NG7 2RD, UK; School of Psychology, University of Nottingham, Nottingham NG7 2RD, UK; School of Psychology, University of Nottingham, Nottingham NG7 2RD, UK; Division of Clinical Neuroscience, University of Nottingham, Nottingham NG7 2UH, UK; Department of Neurology, Nottingham University Hospitals NHS Trust, Queen’s Medical Centre, Nottingham NG7 2UH, UK; Centre for Dementia, Institute of Mental Health, University of Nottingham, Nottingham NG7 2TU, UK; MRC Cognition and Brain Sciences Unit, University of Cambridge, Cambridge CB2 7EF, UK; School of Psychology, University of Nottingham, Nottingham NG7 2RD, UK; Centre for Dementia, Institute of Mental Health, University of Nottingham, Nottingham NG7 2TU, UK; NIHR Biomedical Research Centre, University of Nottingham, Nottingham NG7 2UH, UK

**Keywords:** frontotemporal dementia, behavioural variant FTD, nonfluent variant PPA, semantic dementia, logopenic variant PPA

## Abstract

Frontotemporal dementia (FTD) and primary progressive aphasia (PPA) are complex, partially overlapping neurodegenerative syndromes primarily affecting the frontal and temporal lobes, resulting in deficits in behaviour, executive function and language. FTD is among the leading causes of early-onset dementia and has several subtypes, including behavioural-variant FTD (bvFTD) and PPA. PPA is subdivided into three main variants: non-fluent variant PPA (nfvPPA), semantic variant PPA (svPPA)/semantic dementia (SD) and logopenic variant PPA (lvPPA), each characterized by distinct language and cognitive impairments. Although these syndromes are clinically distinguishable, overlapping cognitive, behavioural and neuroanatomical features are common. To provide a comprehensive quantitative synthesis of the associated neuroimaging findings for each subgroup, we conducted an activation likelihood estimation (ALE) meta-analysis of structural and functional neuroimaging studies across bvFTD and the major PPA syndromes. The analysis included coordinate-based data from 114 studies comprising 8057 patients. Across syndromes, patients showed widespread brain abnormalities relative to healthy controls, involving frontal, temporal and parietal cortices as well as subcortical and limbic regions, including the basal ganglia and thalamus. Each FTD subtype demonstrated distinct, yet partially overlapping, patterns of degeneration. bvFTD showed prominent degeneration in the frontal and medial temporal lobes, insula, cingulate cortex and limbic system, consistent with impairments in social cognition and disinhibition. svPPA/SD exhibited focal atrophy in the anterior temporal lobes, disrupting the semantic network and impairing semantic processing. nfvPPA was associated with degeneration in the speech production network, particularly the insula and inferior frontal gyrus. lvPPA displayed left-lateralized abnormalities in the posterior temporal and inferior parietal lobes, affecting language function. In addition to these prototypical patterns, overlapping regions were observed between specific syndromes, including shared involvement of anterior temporal and limbic regions in bvFTD and svPPA/SD, frontal–insular regions in bvFTD and nfvPPA and lateral temporal regions in svPPA/SD and lvPPA. Together, these findings provide a robust synthesis of distinct and overlapping neuroanatomical alterations across FTD and PPA syndromes, clarifying how syndrome-specific and shared patterns of degeneration may contribute to clinical heterogeneity.

## Teaser

Ilchovska *et al*. performed an activation likelihood estimation meta-analysis of 8057 frontotemporal dementia patients, revealing common and distinct patterns of brain degeneration across four different subtypes. The study concludes that both unique and overlapping neural abnormalities underpin the diverse clinical presentations of frontotemporal dementia.

## Graphical Abstract

**Graphical Abstract:**
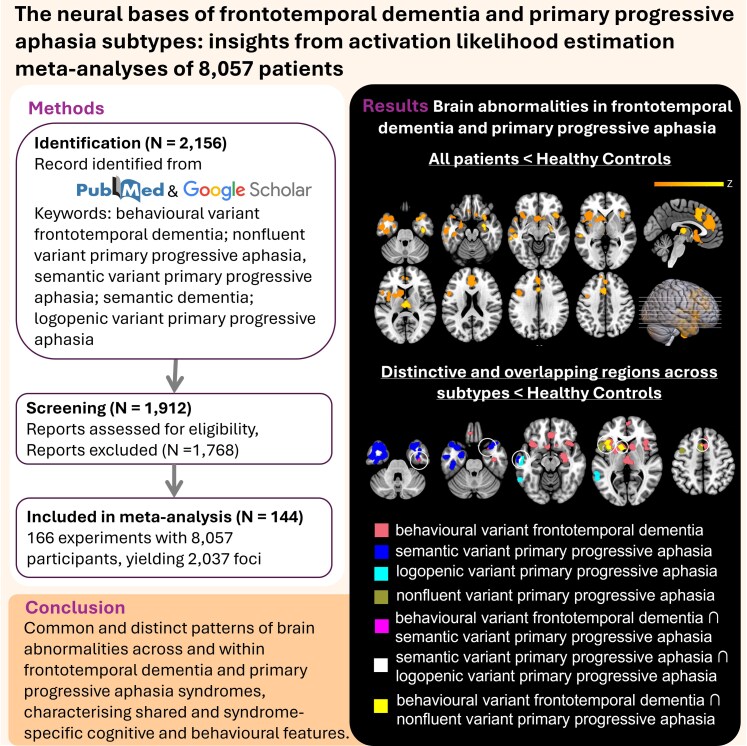

## Introduction

Frontotemporal dementia (FTD) is a neurodegenerative syndrome characterized by the progressive deterioration of the frontal and temporal lobes, resulting in deficits in behaviour, executive function and language.^1-3^ This is the most prevalent type of early-onset dementia and ranks as the third most common form of dementia overall, following Alzheimer's disease and Lewy body disease.^4-6^ Clinically, FTD encompasses various clinical syndromes distinguished by predominant features. Approximately half of cases manifest as the behavioural variant FTD (bvFTD), characterized by disruptions in social behaviour and personality,^7^ while the remainder fall under the label of primary progressive aphasia (PPA), demonstrating language decline.^8^

PPA is conventionally subdivided into three main variants: non-fluent variant PPA (nfvPPA) characterized by speech production difficulties,^9^ semantic variant PPA (svPPA) or semantic dementia (SD) exhibiting multimodal semantic memory degradation,^10^ and logopenic variant PPA (lvPPA) marked by impairments in sentence repetition, word finding and syntactic comprehension.^11^ Although most PPA syndromes are associated with frontotemporal lobar degeneration (FTLD) pathology, lvPPA is more frequently linked to Alzheimer's pathology.^12,13^ In the present study, lvPPA was included to provide a comprehensive synthesis of the major clinical PPA syndromes, while recognizing that its underlying neuropathology often differs from that of FTLD.^14,15^

SD is a clinical syndrome that varies in a graded manner from predominantly language-led features (classical left-lateralized svPPA)^8^ through to more prominent prosopagnosia, behavioural and socio-emotional changes associated with greater right temporal lobe involvement.^16-20^ These right-dominant presentations have been described using several overlapping terms, including right temporal variant FTD, semantic behavioural variant FTD or right-sided semantic dementia.^21^ Given their shared core semantic impairment and frequent reporting as one clinical group (i.e. SD) in the literature,^18^ they were treated as a single entity in the present meta-analysis.

Neuroimaging studies have contributed to advancing our understanding of FTD and PPA (for a review, see^22^). Studies utilizing structural magnetic resonance imaging and fluorodeoxyglucose positron emission tomography (FGD-PET) have revealed distinct patterns of brain atrophy and hypometabolism corresponding to the clinical and pathological variants of FTD and PPA. Diffusion tensor imaging (DTI) and resting-state functional magnetic resonance imaging (rsfMRI) have delineated structural and functional changes in brain connectivity. In bvFTD, brain atrophy impacts various regions including the orbitofrontal cortex (OFC), medial/lateral prefrontal cortex, anterior cingulate cortex (ACC), insula and subcortical areas.^23-25^ White matter damage involves pathways, including the superior longitudinal fasciculus and corpus callosum.^26,27^ Previous studies of bvFTD have found decreased functional connectivity within the salience network, including the prefrontal cortex, insula and ACC.^28-30^ svPPA/SD is linked to atrophy and hypometabolism in the anterior temporal lobe (ATL)^10,31,32^ along with damage to the uncinate fasciculus and the inferior longitudinal fasciculus.^33^ nfvPPA involves atrophy of the left inferior frontal gyrus (IFG), insula and premotor cortex,^34-36^ while lvPPA is associated with atrophy in the left superior temporal and inferior parietal lobe (IPL).^37,38^ These structural and functional alterations are associated with the clinical symptoms of the bvFTD and PPA syndromes.

However, individual neuroimaging studies in FTD and PPA often yield inconsistent findings due to various factors, including small cohort sizes, varying clinical samples, different imaging techniques and methodological variability. Coordinate-based meta-analysis (CBMA) offers a powerful approach to address these limitations by quantitatively synthesizing results across studies, increasing statistical power and identifying robust, reproducible patterns of brain alteration.^39^ Previous CBMA studies in FTD have focused on individual syndromes, demonstrating convergent abnormalities in bvFTD^40^ and svPPA/SD.^41^ However, no study has provided a comprehensive overview of brain alterations across both bvFTD and the different PPAs.

Clinical and pathological heterogeneity within FTD syndromes is well recognized, with substantial variability observed both within and between diagnostic categories.^20,42-46^ While established diagnostic frameworks capture prototypical presentations, many patients exhibit overlapping or mixed features. Rather than replacing existing clinical classifications, recent work has proposed that FTD syndromes may be understood as occupying partially overlapping positions within a multidimensional neurocognitive space, shaped by the distribution of neurodegeneration across large-scale brain networks.^19,20,42,46,47^ From this perspective, classical syndromes represent anchor points within a broader continuum of phenotypic variation. For example, bvFTD and SD/svPPA form a clear frontotemporal continuum associated with behavioural and executive impairment as atrophy affects frontal regions and multimodal semantic impairment resulting from ATL pathology.^19,20,44,47^ Here, we aimed to establish a convergent understanding of the neural basis across the FTD spectrum.

The main goal of this study was to elucidate the neural basis of FTD spectrum through a CBMA of published neuroimaging studies. We assessed the consensus of structural and functional alterations in the bvFTD and svPPA/SD, nfvPPA and lvPPA, employing activation likelihood estimation (ALE) meta-analysis. Specifically, we examined (i) the overall convergence of findings from structural and functional neuroimaging studies, (ii) the concurrence within each FTD/PPA variant, and (iii) the overlaps and distinctiveness between the different FTD/PPA variants. Through this examination, we determined whether structural and functional alterations can be linked to clinical phenotypes in FTD/PPA, contributing to both its heterogeneity and shared characteristics.

## Materials and methods

This large-scale CBMA was conducted in accordance with the latest best-practice guidelines for neuroimaging meta-analyses^39^ and followed the PRISMA (Preferred Reporting Items for Systematic Reviews and Meta-Analyses) statement.^48^ The study protocol was pre-registered on the International Prospective Register of Systematic Reviews (PROSPERO) under the code CRD42023449002.

### Search strategy and criteria

We conducted a PubMed and Google Scholar search on articles published up to Oct 2023 on the four selected clinical types (bvFTD, svFTD/SD, nfvPPA and lvPPA). Our search strategy involved the following combinations of keywords: (behavioural variant frontotemporal dementia OR bvFTD OR frontal variant frontotemporal dementia OR frontotemporal dementia OR Pick's disease OR primary progressive aphasia OR semantic dementia OR semantic variant PPA OR svPPA OR progressive non-fluent aphasia OR agrammatic variant PPA OR nonfluent variant PPA OR nfvPPA OR logopenic primary progressive aphasia phonological variant PPA OR logopenic variant PPA OR lvPPA) AND (functional magnetic resonance imaging OR fMRI OR voxel-based morphometry OR VBM OR positron emission tomography OR PET).

**Figure 1:**
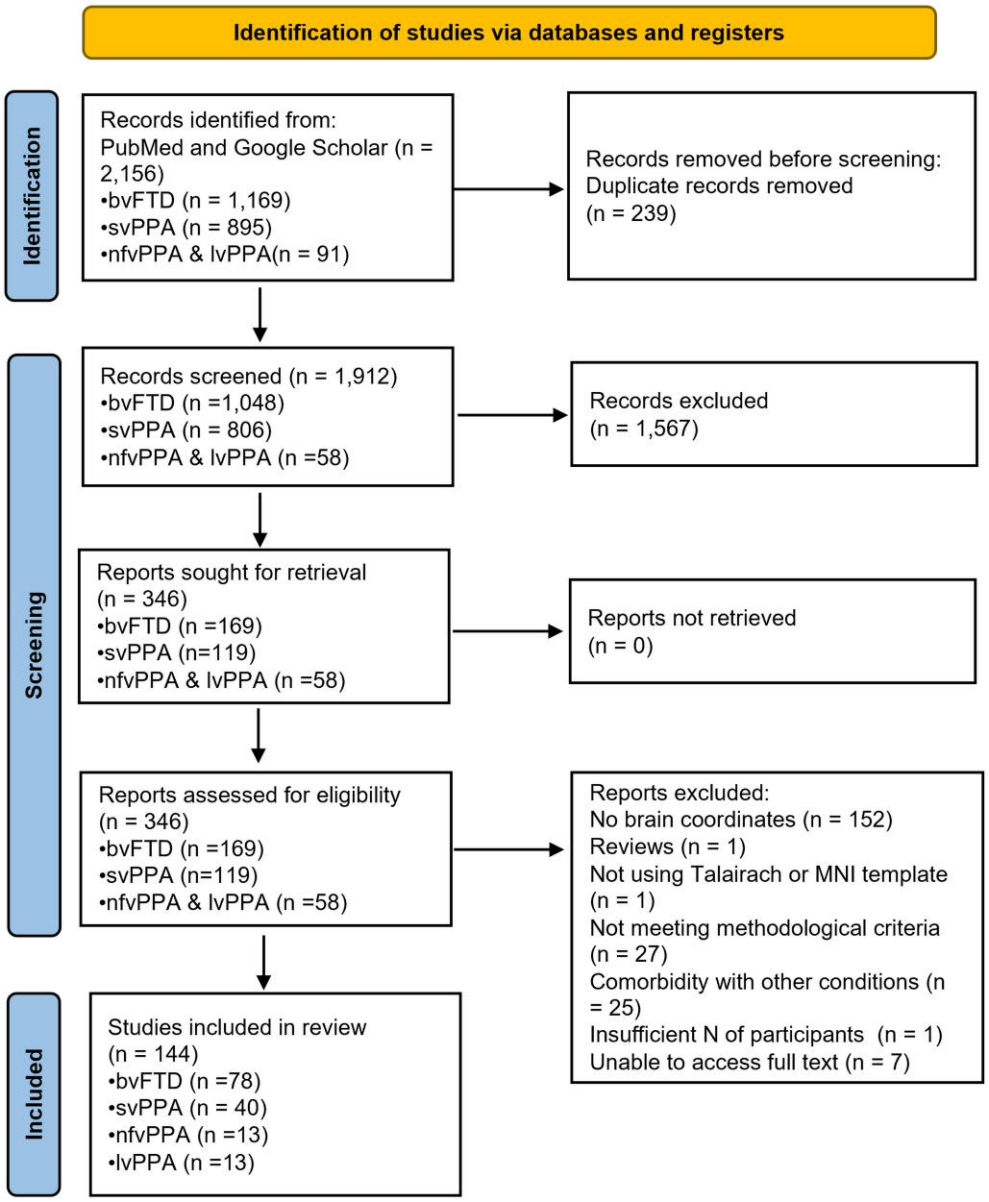
PRISMA (Preferred Reporting Items for Systematic Reviews and Meta-Analyses) flow diagram of the study selection process.

We searched for articles that met the following criteria: (i) published in English; (ii) contained a between-group comparison of a healthy control (HC) group and clinically diagnosed FTD/PPA patients, where the patient group has no concurrent psychiatric disorder, other forms of dementia, neurological symptoms or any history of substance abuse; (iii) included a minimum of six participants per group; (iv) employed task-based fMRI, resting-state fMRI, VBM or FDG-PET neuroimaging techniques for data collection; (v) performed a whole-brain analysis; (vi) reported brain coordinates in Talairach or Montreal Neurological Institute (MNI) standard space; (vii) for structural imaging studies, reported grey matter coordinates (excluding white matter coordinates). We excluded studies using seed-based functional connectivity analysis, DTI or cortical thickness measurements. After removing duplicates, three authors (Z.I, J.L. and E.P.) independently reviewed the unique records, initially by screening abstracts and subsequently by reading the full texts of potentially relevant articles. Any disagreements among the three raters were resolved by an additional reviewer (J.J.). Figure 1 illustrates a flow diagram based on the PRISMA guidelines, illustrating the inclusion and exclusion criteria applied during the study selection process.

Sex information was not consistently reported across all included studies and therefore could not be systematically incorporated into the meta-analysis. Where sex data were available, reporting was heterogeneous and incomplete. For example, across a subset of studies with extractable data, the sample included 189 females and 219 males; however, these values are not representative of the full dataset.

### Activation likelihood estimation analysis

The updated ALE algorithm using Ginger ALE (version 3.0.2) was employed to detect convergent patterns of brain alterations, demonstrating that the convergence of reported coordinates across experiments exceeds what would be expected from random spatial associations.^49^ This method tests the spatial convergence of reported coordinates for differences between patients and healthy controls (HCs) against the null hypothesis of randomly distributed findings across the brain. Each experiment's foci were modelled as 3D Gaussian kernels to represent the uncertainty about the peak coordinates’ locations. A modelled activation map was then created for each experiment by combining all the convolved foci. The union of these maps for all included experiments resulted in an ALE score map, representing the voxel-wise likelihood of convergent findings at each brain location. To distinguish true convergence from random overlap, the ALE score map was statistically tested against a null distribution of randomly distributed findings, with a *P* < 0.05 cluster-level family-wise error (cFWE) correction for multiple comparisons (a cluster-forming *P* < 0.001 with thresholding 1000 permutations). Prior to the analysis, all Talairach coordinates were transformed into MNI space using the transform called icbm2tal developed by Laird *et al*.^50^

To test the spatial convergence of reported coordinates for differences between patients and HCs, we performed separate ALE meta-analyses for each clinical subtype: (i) clinical subtype < HCs (structural and functional imaging); (ii) clinical subtype < HCs (structural imaging); (iii) clinical subtype < HCs (functional imaging: FDG-PET, rsfMRI and task-fMRI experiments), (iv) clinical subtype > HCs (structural and functional imaging); (v) clinical subtype > HCs (structural imaging); (vi) clinical subtype > HCs (functional imaging). We performed conjunction and contrast analyses to identify overlapping and distinctive brain atrophy and functional alterations between different bvFTD and PPA syndromes. The ALE score map was statistically tested against a null distribution of randomly distributed findings, with a *P* < 0.05 cluster-level family-wise error (cFWE) correction for multiple comparisons (a cluster-forming *P* < 0.001 with thresholding 1000 permutations).

## Results

### Convergent regional abnormalities across all clinical subtypes

**Figure 2:**
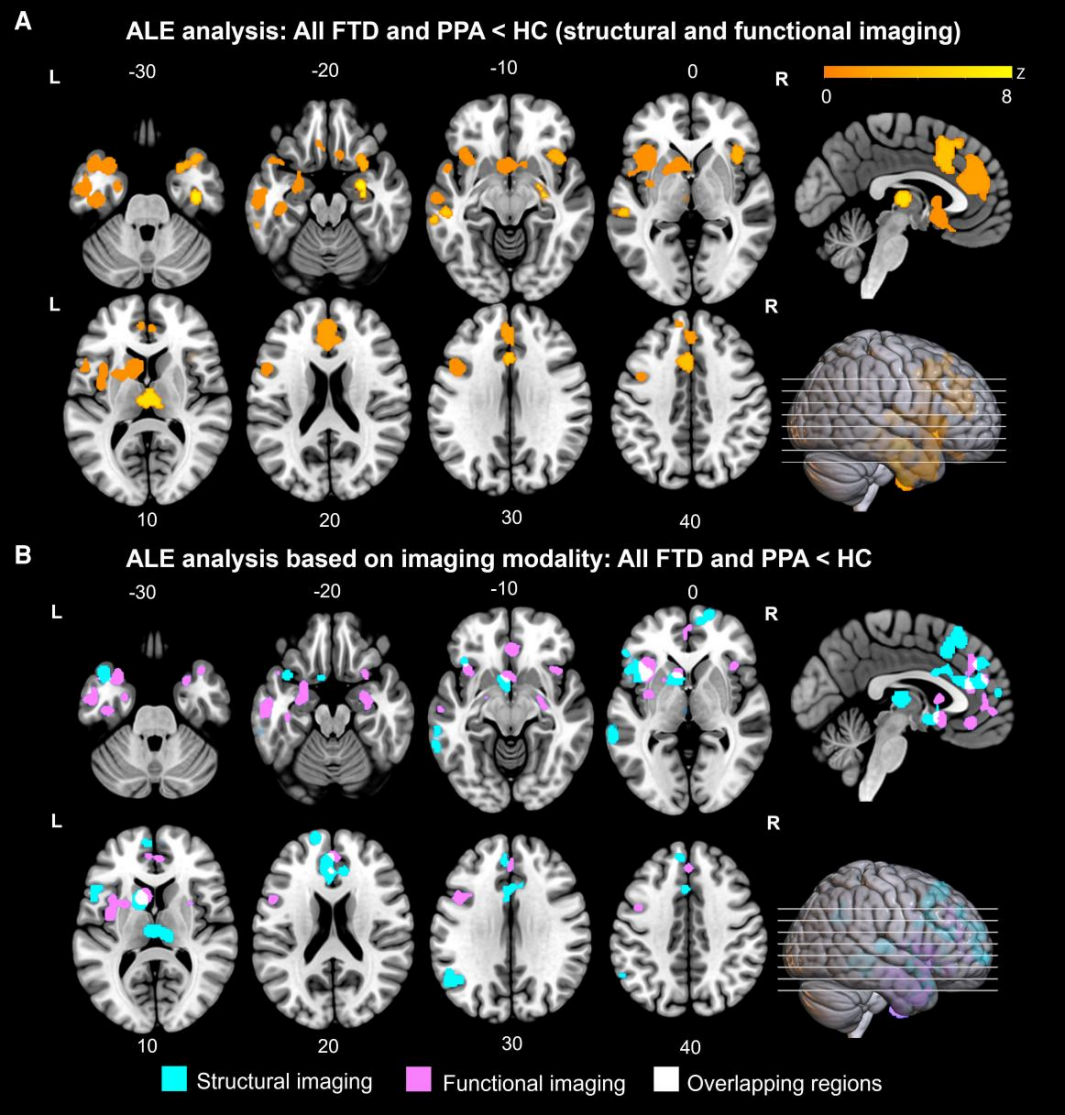
**Convergent brain abnormalities in FTD and PPA syndromes compared to healthy controls.** (**A**) Experiments reporting atrophy/hypoactivation. (**B**) Experiments using structural (green) and functional (red) modalities, with overlapping regions shown in yellow. Activation likelihood estimation (ALE) meta-analysis was performed using a random-effects model with cluster-level family-wise error correction (cFWE *P* < 0.05), using a cluster-forming threshold of *P* < 0.001 and 1000 permutations. All clusters were significant at cFWE *P* < 0.05. ALE values reflect the convergence of reported coordinates across experiments rather than conventional test statistics (e.g. *t* or *F* values). The analysis included 166 experiments (*N* = 8057 participants; structural: 118 experiments, *n* = 6179; functional: 48 experiments, *n* = 1878). The experimental unit corresponds to independent neuroimaging experiments extracted from published studies. Coordinates are presented in Montreal Neurological Institute (MNI) space. Colour bars represent *Z* values. Abbreviations: FTD = frontotemporal dementia; PPA = primary progressive aphasia; MNI = Montreal Neurological Institute.

A total number of 144 studies involving 166 experiments with 8057 participants and yielding 2037 foci were included in our meta-analysis. The results are summarized in Fig. 2 and Supplementary Table 1.

First, we examined the consensus of structural and functional abnormalities by pooling all studies. In contrast to all subtypes < HC, we identified eight convergent clusters in the caudate, anterior cingulate cortex (ACC), insula, inferior frontal gyrus (IFG), superior temporal gyrus (STG), middle temporal gyrus (MTG), inferior temporal gyrus (ITG), cingulate cortex (CC), superior frontal gyrus (SFG), thalamus, parahippocampal gyrus (PhG), amygdala and hippocampus (Fig. 2A).

Next, we performed separate ALE analyses for each imaging modality. The ALE analysis of the structural imaging data (foci = 1390, experiments = 118, subjects = 6179) revealed nine significant clusters in the STG, MTG, PhG, insula, caudate, IFG and ACC (Fig. 2B). The results from functional imaging (foci = 647, experiments = 48 and subjects = 1878) showed 10 significant clusters in the ACC, SFG, medial frontal gyrus (MedFG), IFG, insula, thalamus, caudate, STG and MTG (Fig. 2B). There were no significant clusters identified in all subtypes > HC comparisons.

### Regional abnormalities in each clinical subtype

**Figure 3:**
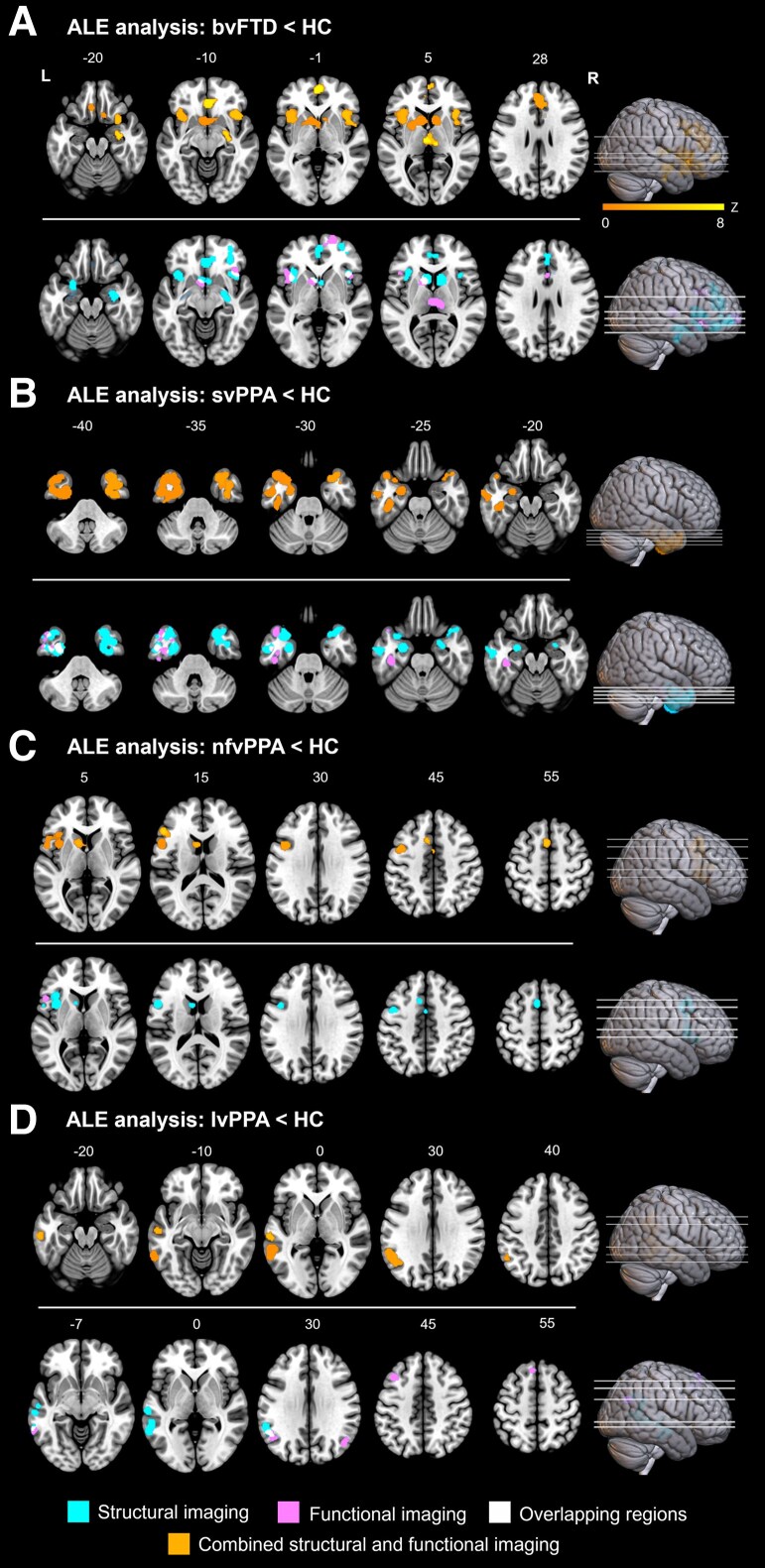
**Convergent brain abnormalities in the bvFTD and PPA syndromes compared to healthy controls.** (**A**) The results of ALE analysis in the contrast of bvFTD < HC. (**B**) The results of ALE analysis in the contrast of svPPA/SD < HC. (**C**) The results of ALE analysis in the contrast of nfvPPA < HC. (**D**) The results of ALE analysis in the contrast of lvPPA < HC. Experiments using structural (green) and functional (red) modalities, with overlapping regions shown in yellow. ALE meta-analyses were conducted using a random-effects model with cluster-level family-wise error correction (cFWE *P* < 0.05; cluster-forming threshold *P* < 0.001; 1000 permutations). All clusters were significant at cFWE *P* < 0.05. ALE values reflect spatial convergence across studies rather than conventional test statistics. Sample sizes: bvFTD (94 experiments, *n* = 4508), svPPA/SD (41 experiments, *n* = 1818), nfvPPA (17 experiments, *n* = 855), lvPPA (14 experiments, *n* = 876). The experimental unit corresponds to independent neuroimaging experiments. Coordinates are presented in Montreal Neurological Institute (MNI) space Colour bars represent Z values. Abbreviations: bvFTD = behavioural variant frontotemporal dementia; svPPA = semantic variant primary progressive aphasia; SD = semantic dementia; nfvPPA = nonfluent variant primary progressive aphasia; lvPPA = logopenic variant primary progressive aphasia; HC = healthy controls; MNI = Montreal Neurological Institute.

To identify convergent brain abnormalities for the bvFTD and PPA syndromes, we conducted separate ALE analyses for each subtype (Fig. 3 and Supplementary Tables 2–5). No significant clusters were found in each clinical subtype > HC comparisons.

For the bvFTD < HC contrast (foci = 1190, experiments = 94, subjects = 4508), nine clusters were identified, including the caudate, putamen, MedFG, thalamus, ACC, cingulate cortex (CC), insula, IFG, STG, amygdala, hippocampus and fusiform gyrus (FG) (Fig. 3A, top). The structural imaging comparison of bvFTD < HC (foci = 831, experiments = 68, subjects = 3490) revealed 10 significant clusters, comprising the MedFG, ACC, insula, amygdala, hippocampus, FG, IFG, caudate and PhG (Fig. 3A, bottom). For the functional imaging comparison of bvFTD < HC (foci = 370, experiments = 26, subjects = 1018), six significant clusters were found in the caudate, thalamus, insula, MedFG and CC (Fig. 3A, bottom). The results are summarized in Supplementary Table 2.

In contrast to svPPA/SD < HC (foci = 349, experiments = 41 and subjects = 1818), we found two clusters in bilateral anterior temporal lobes (ATL) covering the STG, MTG and ITG (Fig. 3B, top). The structural imaging comparison of svPPA/SD < HC (foci = 219, experiments = 29 and subjects = 1422) revealed four clusters in the STG, MTG, ITG and PhG, while the functional imaging comparison (foci = 130, experiments = 12 and subjects = 396) showed a cluster in the left ATL (Fig. 3B, bottom). The results are summarized in Supplementary Table 3.

In nfvPPA, the contrast of nfvPPA < HC (foci = 228, experiments = 17 and subjects = 855) revealed five clusters in the IFG, insular, precentral gyrus, caudate, MedFG, CC and MFG (Fig. 3C, top). The structural imaging comparison of nfvPPA < HC (foci = 183, experiments = 12 and subjects = 678) identified five clusters in the insula, IFG, precentral gyrus, MedFG, CC, SFG and caudate. The functional imaging comparison (foci = 45, experiments = 5 and subjects = 177) revealed clusters only in the IFG and precentral gyrus (Fig. 3C, bottom). The results are summarized in Supplementary Table 4.

In lvPPA, the contrast of lvPPA < HC (foci = 259, experiments = 14 and subjects = 876) identified four clusters in the left STG, MTG, ITG, supramarginal gyrus and inferior parietal lobe (IPL) (Fig. 3D, top). The subsequent structural imaging comparison (foci = 157, experiments = 9 and subjects = 589) revealed three clusters including the STG, MTG, ITG, supramarginal gyrus and inferior parietal lobe in the left hemisphere (Fig. 3D, bottom). Functional imaging analysis (foci = 102, experiments = 5, subjects = 287) demonstrated five clusters in the left temporal lobe and frontal lobe (Fig. 3D, bottom). The results are summarized in Supplementary Table 5.

### Distinctive and overlapping regions of the clinical subtypes

**Figure 4:**
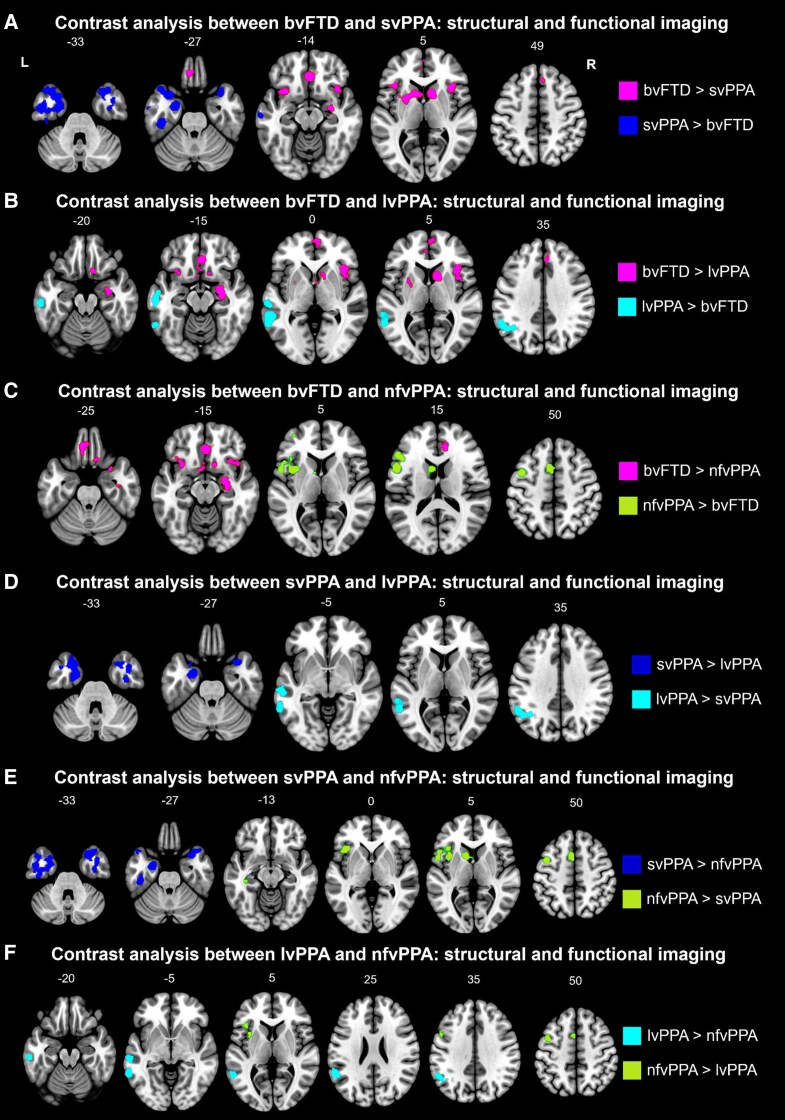
**Distinctive brain abnormalities across FTD and PPA syndromes.** (**A**) The results of contrast analysis between bvFTD and svPPA/SD. (**B**) The results of contrast analysis between bvFTD and lvPPA. (**C**) The results of contrast analysis between bvFTD and nfvPPA. (**D**) The results of contrast analysis between svPPA/SD and lvPPA. (**E**) The results of contrast analysis between svPPA/SD and nfvPPA. (**F**) The results of contrast analysis between lvPPA and nfvPPA. ALE meta-analyses were conducted using a random-effects model with cluster-level family-wise error correction (cFWE *P* < 0.05; cluster-forming threshold *P* < 0.001; 1000 permutations). All clusters were significant at cFWE *P* < 0.05. ALE values reflect spatial convergence across studies rather than conventional test statistics. Sample sizes: bvFTD (94 experiments, *n* = 4508), svPPA/SD (41 experiments, *n* = 1818), nfvPPA (17 experiments, *n* = 855), lvPPA (14 experiments, *n* = 876). The experimental unit corresponds to independent neuroimaging experiments. Coordinates are presented in Montreal Neurological Institute (MNI) space. Abbreviations: bvFTD = behavioural variant frontotemporal dementia; svPPA = semantic variant primary progressive aphasia; SD = semantic dementia; nfvPPA = nonfluent variant primary progressive aphasia; lvPPA = logopenic variant primary progressive aphasia.

We further investigated the distinctive and overlapping patterns of brain abnormalities in the clinical subtypes by conducting contrast analyses between pairs of subtypes. When comparing bvFTD with svPPA/SD, bvFTD exhibited distinct abnormalities in regions such as the caudate, ACC, MedFG, SFG, insula, IFG and parahippocampal gyrus, whereas svPPA/SD showed focalized abnormalities primarily in the bilateral ATLs (Fig. 4A). In the comparison between bvFTD and lvPPA, similar regions were affected in bvFTD, including the caudate, parahippocampal gyrus, insula, IFG, ACC, MedFG and putamen, while lvPPA demonstrated distinctive abnormalities in the left posterior STG, MTG, ITG, supramarginal gyrus and IPL (Fig. 4B). In the analysis between bvFTD and nfvPPA, bvFTD was associated with abnormalities in the parahippocampal gyrus, insula, IFG, ACC and MedFG, whereas nfvPPA showed changes in the left IFG, insula, precentral gyrus, MedFG, MFG and caudate (Fig. 4C). When comparing svPPA/SD with other PPA subtypes, svPPA/SD exhibited limited abnormalities, primarily in the bilateral ATLs (Fig. 4D and E). In contrast, lvPPA relative to svPPA/SD showed greater left-lateralized abnormalities in the posterior STG, MTG, ITG, supramarginal gyrus and IPL (Fig. 4D), while nfvPPA, compared to svPPA/SD, showed distinct changes in the left IFG, insula, precentral gyrus, MFG, MedFG, SFG and CC (Fig. 4E). In the comparison between lvPPA and nfvPPA, lvPPA demonstrated additional abnormalities in the left supramarginal gyrus, IPL and posterior temporal lobe, whereas nfvPPA exhibited changes in the left MedFG, SFG, MFG, IFG, precentral gyrus and insula (Fig. 4F). Overall, bvFTD displayed unique brain abnormalities in regions including the bilateral IFG, insula, MedFG, ACC and caudate when compared with PPAs. svPPA/SD was characterized by distinct abnormalities in the bilateral ATLs relative to other subtypes. lvPPA showed unique changes in the left posterior temporal lobe, supramarginal gyrus and IPL, while nfvPPA presented distinctive abnormalities in the left IFG, insula, precentral gyrus, MFG and MedFG. These findings are summarized in Fig. 4 and Supplementary Tables 6–11.

**Figure 5:**
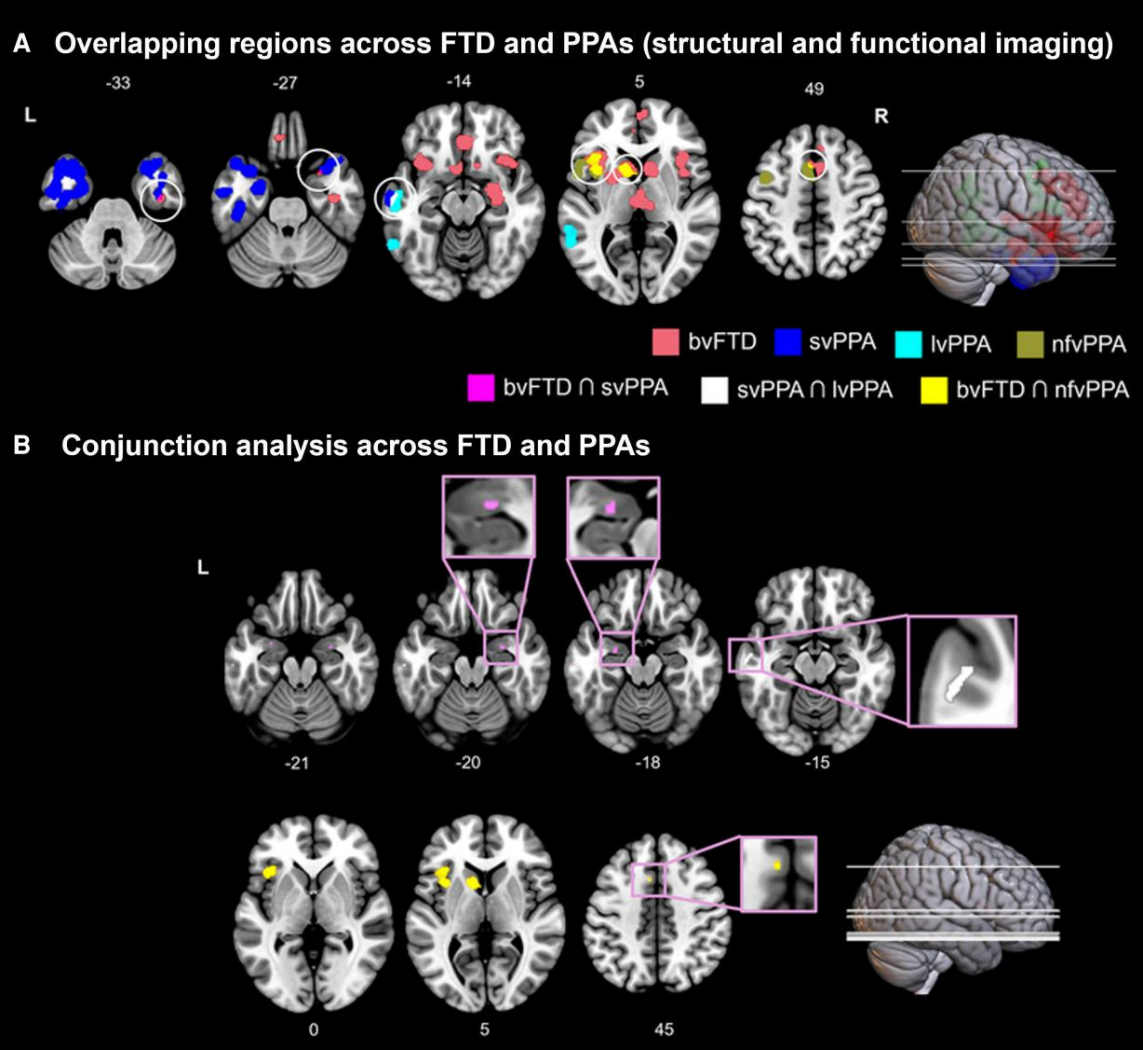
**Common regions across FTD and PPA syndromes.** (**A**) Overlapping regions across FTD subtypes. (**B**) The results of conjunction analysis across the FTD subtypes. ALE meta-analyses were conducted using a random-effects model with cluster-level family-wise error correction (cFWE *P* < 0.05; cluster-forming threshold *P* < 0.001; 1000 permutations). All clusters were significant at cFWE *P* < 0.05. ALE values reflect spatial convergence across studies rather than conventional test statistics. Sample sizes: bvFTD (94 experiments, *n* = 4508), svPPA/SD (41 experiments, *n* = 1818), nfvPPA (17 experiments, *n* = 855), lvPPA (14 experiments, *n* = 876). The experimental unit corresponds to independent neuroimaging experiments. Coordinates are presented in Montreal Neurological Institute (MNI) space. Abbreviations: bvFTD = behavioural variant frontotemporal dementia; svPPA = semantic variant primary progressive aphasia; SD = semantic dementia; nfvPPA = nonfluent variant primary progressive aphasia; lvPPA = logopenic variant primary progressive aphasia.

We overlapped ALE results from each type (combined structural and functional imaging) and found overlapping regions between the clinical subtypes (Fig. 5A). Specifically, bvFTD and svPPA/SD showed an overlap in the right ventral ATL, svPPA/SD and lvPPA overlapped in the left MTG, and bvFTD and nfvPPA overlapped in the left insula, caudate and ACC. To confirm these findings, we performed conjunction analysis across the FTD subtypes. Conjunction analyses revealed that bvFTD and svPPA/SD overlapped in the bilateral amygdala (in structural imaging only). Additionally, svPPA/SD and lvPPA shared a common region in the left MTG, while bvFTD and nfvPPA shared abnormalities in the left insula, IFG, caudate and MedFG. Notably, no regions were found to overlap across three or four subtypes. These results were summarized in Fig. 5B and Supplementary Tables 6–11.

## Discussion

To understand the neural bases of the FTD/PPA spectra, we performed a large-scale CBMA on both structural and functional brain studies across the bvFTD and PPA syndromes compared to healthy individuals. We analysed neuroimaging data from 8057 FTD patients, including those with bvFTD, svPPA/SD, nfvPPA and lvPPA, making this the largest ALE meta-analysis on this topic (*N* = 1261).^51^ Rather than supporting a view of entirely distinct syndromes or a fully continuous pattern of degeneration, our findings reveal that these clinical entities are associated with robust, prototypical patterns of brain abnormality alongside reproducible areas of overlap between specific syndromes. Across all groups, FTD and PPA syndromes showed widespread involvement of frontal, temporal and parietal cortices, as well as subcortical and limbic structures. These findings are consistent with the broad range of behavioural, executive and language impairments observed clinically. Importantly, each syndrome exhibited a characteristic neuroanatomical signature that aligns closely with established diagnostic criteria, reinforcing the validity of current clinical classifications. At the same time, the meta-analysis identified circumscribed regions of shared degeneration between specific syndromes, reflecting known areas of clinical overlap. These shared patterns did not converge on a single common region across all syndromes but instead revealed selective overlaps that may contribute to overlapping cognitive and behavioural features.^19,20,42,44,45,47^ This combination of distinctiveness and convergence supports the view that FTD and PPA syndromes occupy partially overlapping positions within a graded, multidimensional neurocognitive landscape, rather than forming strictly isolated or fully homogeneous categories.^18,42,44-46^

### Distinctive patterns of brain abnormalities in each syndrome

FTD and PPA patients overall were more likely to exhibit both structural and functional brain abnormalities in various regions of the frontal, temporal and parietal lobes, as well as in the basal ganglia and limbic systems, compared to healthy controls. This widespread degeneration across brain systems aligns with the heterogeneous clinical symptoms and cognitive impairments observed in FTD/PPA, including deficits in behaviour, executive function, language and emotional processing.^1-3^ Our findings are consistent with the established neural characteristics of each clinical subtype and their associated core symptoms.

bvFTD was characterized with degeneration in the salience network (insular, ACC and CC), limbic system (caudate and thalamus), frontal lobe (SFG, IFG and MedFG) and medial temporal lobe (amygdala, hippocampus and parahippocampus). Compared to other clinical subtypes, bvFTD showed distinct abnormalities in the caudate, ACC, insula, medFG, IFG and right parahippocampus (Fig. 4). Atrophy and hypoactivation/disconnection in these regions are closely associated with the core diagnostic symptoms bvFTD, including impaired empathy, diminished social awareness and reduced impulse control. Early degeneration of Von Economo neurons (VENs) in the ACC, essential for empathy, social awareness and self-control, plays a significant role in bvFTD's social and emotional impairments.^52^ VENs in the anterior insular, another key region in awareness, form a functional network with the striatum and amygdala.^53^ Together, the ACC and insular are central to the salience network, guiding behaviour by assessing the relevance of both internal and external events.^54^ The amygdala, crucial for emotional learning and behaviour, is also affected early in bvFTD.^55^ Degeneration in the frontal lobe, responsible for self-awareness, cognitive control, emotion regulation and impulse control, contributes to the disinhibition commonly observed in bvFTD.^24^ Key neural foundations of empathy like the anterior insula, amygdala, ACC, thalamus and lateral frontal regions,^56^ are also impacted, further explaining bvFTD's characteristic social and emotional deficits.^57^ The extensive degeneration in these regions results in hallmark bvFTD symptoms such as impaired emotional processing, social cognition deficits, disinhibition, executive dysfunction and apathy.^40^ Additionally, impaired episodic memory in bvFTD is often due to executive dysfunction, which affects the ability to monitor and organize memories.^58^ Similar to Alzheimer's disease (AD), amnestic bvFTD patients exhibit atrophy or dysfunction in the hippocampus and other paraphippocampus.^59^

Very different from other subtypes, svPPA/SD showed brain atrophy, hypoactivation/decreased connectivity in the bilateral ATL, including STG, MTG, ITG, FG and paraphippocampal gyrus.^10,60,61^ In svPPA/SD, the defining symptoms include impaired naming and multimodal semantic impairment, accompanied by specific issues such as impaired knowledge about objects and people, and surface dyslexia and dysgraphia, with intact repetition and preserved speech fluency despite lacking specific content.^61^ The core symptoms of svPPA/SD directly stem from ATL degeneration, resulting in progressive semantic degradation across a wide range of concepts, affecting both expressive and receptive language and nonverbal functions. This pattern of semantic deficits is evident across modalities, impacting comprehension and production consistently, a hallmark that distinguishes svPPA/SD from other types.^62,63^ Neuroimaging studies have shown a direct correlation between the extent of semantic deficits and the degree of ATL atrophy and hypometabolism, highlighting the role of the ATLs in svPPA/SD's characteristics.^18,20,64,65^ These findings support the proposal that the ATLs serve as a transmodal transtemporal semantic hub, integrating information from various modality-specific regions across the cortex.^66,67^ This hub synthesizes multimodal inputs over time and context to create coherent, generalized concepts, essential for comprehensive semantic representation.^67,68^ Such integration is critical for maintaining semantic knowledge across sensory and cognitive domains, explaining the extensive semantic degradation seen in svPPA/SD.^68^

nfvPPA exhibited left-lateralized degeneration in the speech production network (SPN), including the insula, IFG, SFG, precentral gyrus and caudate.^69^ This degeneration pattern results in significant speech production difficulties, with patients displaying slow, effortful speech often marked by grammatical errors and impaired motor planning.^9^ In nfvPPA, the left anterior insula, essential for preparing motor actions in speech, like coordinating vocal tract movements,^70^ also showed degeneration.^36^ A longitudinal study has suggested that atrophy in nfvPPA generally begins in the IFG and progressively impacts other structurally and functionally connected regions within the SPN.^69^ Further supporting this, recent resting-state fMRI findings reveal a substantial loss of connectivity in the left IFG,^71^ contributing to motor speech and syntactic impairments.^72^

lvPPA was marked by left-lateralized brain abnormalities in the language network, particularly in the posterior temporal and inferior parietal lobes.^73^ Patients with lvPPA typically have slow, hesitant speech with frequent pauses, use simple but grammatically correct sentences and struggle with naming and repetition tasks.^37^ Damage in these regions disrupts verbal working memory, impairing the ability to retain verbal information as observed in sentence repetition and comprehension tasks.^74,75^ Our results showed that functional imaging meta-analysis revealed degeneration in the left frontal lobe and bilateral inferior parietal regions. A recent study^73^ suggested that the left posterior temporo-parietal cortex is an epicentre of lvPPA, linked to broader connections in the left frontal and temporal language network, which impacts sentence repetition and naming abilities.

Together, these distinct patterns of degeneration in PPAs suggest how different areas of the brain's language network contribute to specific language and speech impairments across PPA subtypes. Our findings indicate that frontal lobe damage mainly impacts phonology and speech fluency, dorsal-posterior temporal lobe lesions affect repetition/verbal working memory and lateral/anterior temporal lobe damage impairs semantic representation.^76^

### Overlapping brain abnormalities between FTD syndromes

Recent research has proposed that the behavioural changes observed in FTD result from disruptions to large-scale brain networks.^77,78^ A neurocognitive model has been proposed to explain these impairments. The controlled social-semantic cognition (CS-SC) model offers a unified frontotemporal framework for understanding social dysfunction across FTD subtypes.^19,20,44^ In this model, social impairments in FTD arises from disruption to two interacting systems: (i) social-semantic knowledge, supported by the ATL and (ii) social control processes such as selection, evaluation, decision-making and inhibition, mediated by the frontal cortices, particularly the OFC and medial/lateral prefrontal regions.^19^ This framework helps explain both the semantic and social deficits seen in bvFTD and svPPA/SD, emphasizing the interaction between prefrontal and temporal regions in guiding social behaviour.

A related network-based perspective implicates disruptions to broader large-scale systems such as the semantic appraisal network (SAN).^77^ Within the SAN, the ATL serves as a core hub for representing social-semantic knowledge, while the amygdala and ventromedial OFC contribute to tagging this information with emotional value. Damage to this network can strip social concepts of their meaning and emotional salience, leading to the socioemotional deficits commonly observed in FTD. Recent work also suggests that loss of social-semantic knowledge, particularly following ATL atrophy, may contribute to behavioural disinhibition frequently observed in these patients.^79^

Consistent with these models, our findings revealed that bvFTD and svPPA/SD share overlapping abnormalities in the ATL and bilateral amygdala. In bvFTD, amygdala atrophy is associated with deficits in decision-making, emotional learning and behaviour,^55^ as well as difficulties in reading facial emotions.^80^ Similarly, early amygdala atrophy in svPPA contributes to impairments in emotional processing, reflecting the shared symptoms between these subtypes.^81,82^ As a key node within the SAN,^77^ the amygdala plays a critical role in emotion recognition, integrating socioemotional, interoceptive and episodic information through connections with the ATL and OFC,^83^ highlighting its significance in the socioemotional dysfunction observed across the FTD spectrum.^84^

Our finding also revealed the overlapping degeneration between bvFTD and nfvPPA in the left insula, IFG, MedFG and caudate, key regions supporting controlled selection, inhibition and monitoring of language output.^85^ Language impairments in bvFTD may be driven in part by executive control deficits in frontal regions involved in regulating semantic retrieval and social communication.^19^ As a results, bvFTD patients can display lexico-semantic deficits, impaired expressive prosody and difficulties with reading and writing, while motor speech and grammar often remain relatively preserved.^86-88^ These language impairments tend to worsen with increasing frontotemporal atrophy and may further exacerbate behavioural disturbances in bvFTD.^86^

Both svPPA/SD and lvPPA shared abnormalities in the left lateral temporal lobe, particularly in the MTG. Data-driven analyses have identified two subgroups within the lvPPA: one displaying prominent naming and object knowledge impairments associated with lateral temporal hypometabolism^89^ and atrophy.^90^ Naming impairments in lvPPA have been linked to abnormalities in the left MTG, suggesting its role in the language dysfunction common to both svPPA/SD and lvPPA.^91^ Importantly, lvPPA patients also exhibit semantic control deficits driven by posterior lateral temporal degeneration, contrasting with the more fundamental semantic degradation characteristic of svPPA/SD. A recent study demonstrated that lvPPA patients show variable semantic performance, with impairments emerging on more demanding tasks (e.g. alternative object use, synonym judgement) while performance remains intact on less challenging semantic tests.^47^

In addition to the existing diagnostic frameworks that provide paradigmatic prototypical descriptions of each clinical subtype, our results provide neuroanatomical support for the long-recognized clinical observation that overlap between FTD syndromes can arise when degeneration affects shared large-scale networks.^19,77,78^ Multidimensional graded models,^42^ the CS-SC framework^66^ and network-based accounts of FTD^77^ all offer useful conceptual tools for interpreting these overlaps, particularly in relation to shared frontotemporal and limbic involvement. Within this context, our findings help delineate where, and between which syndromes, such overlap is most consistently observed.

Together, these results highlight that while FTD and PPA syndromes are clinically and neuroanatomically distinguishable, they are not entirely discrete. Instead, individual patients express mixed or evolving phenotypes depending on the distribution and progression of neurodegeneration across interconnected brain networks. Our meta-analytic synthesis provides a quantitative reference for these patterns, complementing existing clinical frameworks rather than replacing them.

### Limitations and future directions

Despite its size and robust results, this study has limitations. First, a significant constraint of our meta-analysis is the inherent inability to integrate individual-level genetic, fluid biomarker and pathological data. This precludes us from directly correlating specific atrophy or functional connectivity patterns with underlying molecular aetiologies or definitive post-mortem diagnoses. While our study provides a valuable neuroanatomical synthesis, future multi-modal studies that include these measures at the individual patient level will be crucial for a more mechanistic understanding. Second, there was an imbalance in the number of studies per clinical subtype and imaging modality, with relatively low functional imaging studies. This may have influenced the detection and definition of functional clusters, potentially leading to an underrepresentation of functional network alterations. Third, this study did not account for disease stage or severity among participants. FTD/PPA syndromes progress differently across subtypes, with some showing more rapid functional and structural decline. Omitting these factors could obscure potential distinctions or overlaps between subtypes that may only emerge or become more pronounced at specific disease stages. A further limitation is that sex information was inconsistently reported across studies, preventing formal analysis of sex-related effects. Although some studies included sex data, reporting was incomplete and heterogeneous. Future research should prioritize increasing the number of functional imaging studies across clinical subtypes, utilizing advanced imaging techniques like multi-band and multi-echo sequences to capture whole-brain data with high spatial and temporal resolution. These methods enhance sensitivity to subtle functional changes, allowing for better identification of distinct and overlapping networks across subtypes.^92^ Furthermore, longitudinal imaging studies would be beneficial for tracking the progression of abnormalities in both structural and functional networks, providing insight into how degeneration evolves within and across clinical syndromes. By integrating multimodal approaches, including structural, functional and biochemical imaging, future studies could yield a more comprehensive understanding of the dynamic and multifaceted nature of FTD and PPA.

## Supplementary Material

fcag223_Supplementary_Data

## Data Availability

The data used in this meta-analysis were extracted from previously published, peer-reviewed neuroimaging studies. As the analysis is based on aggregate coordinate-based data reported in these studies, no individual participant data were collected or generated for this work. Any additional information related to the meta-analytic dataset is available from the corresponding author upon reasonable request.
